# 

**Published:** 1899-12

**Authors:** 


TABLE OF CONTENTS ON LAST ADVERTISING PAGE.

Vol. XV. DECEMBER, 1899. No. 6.

LIBRARY [illegible]

### TEXAS Medical Journal.

Subscription, $1.00 a Year, in Advance.
Single Copies, 10 Cents.

PUBLISHED AT AUSTIN, TEXAS, BY

F. E. DANIEL, M. D., & S. E. HUDSON, M. D.,

Proprietors.

Eastern Representative: John Guy Monihan, St. Paul Building, 220 Broadway, New York City.

Entered at the Postoffice at Austin, Texas, as Second-class Mail Matter.

